# Implementation and outcome of an electronic tool for detection of paracetamol overdose in a tertiary care hospital

**DOI:** 10.1007/s11096-020-01182-2

**Published:** 2020-10-29

**Authors:** Francisco Cabrera-Diaz, Claudia Zaugg, Silke Lim, Kim Blum, Ali Reza Salili

**Affiliations:** 1 Hospital Pharmacy, Kantonsspital Aarau, 5000 Aarau, Switzerland; 2 Department of Internal Medicine, Clinical Pharmacology, Kantonsspital Aarau, 5000 Aarau, Switzerland

**Keywords:** Alert, Clinical decision support system, Drug related problem, Electronic tool, Medication error, Paracetamol

## Abstract

*Background* Paracetamol is a widely used analgesic and antipyretic drug in hospitals. The development and implementation of an electronic tool with algorithm-based alerts (e-agent) in a clinical information system could reduce the risk of overdose. *Objective* In this study, the performance of such an e-agent developed to detect paracetamol overdosing was analyzed. *Setting* Swiss tertiary care hospital. *Method* All patients ≥ 18 years old who had documented paracetamol administration in the used clinical information system during 2017 were retrospectively screened for an absolute and relative overdosing of paracetamol (> 4 g and > 60 mg/kg/24 h, respectively). This was compared with the patients for which the e-agent had, during the same period, prospectively made an alert for absolute or relative overdosing or for a dosing interval < 4 h (potentially leading to an absolute overdose). *Main outcome measure* E-agent performance defined as detection rate. *Results* of the 13,196 adult patients who received at least one dose of paracetamol, 2292 were exposed at least once to > 4 g/day (17.4%), 39 of these (0.3% of total) were given > 5 g paracetamol. None received more than 6 g. The e-agent detected 87.2% of cases with doses > 5 g. In most cases (87.9%), the cause of the absolute overdose was a switch from intravenous to oral paracetamol, resulting in an absolute overdose the day of the change. The maximal daily dose of 60 mg/kg was exceeded in 30.1% of patients weighing < 50 kg, as well as in 42.3% of patients weighing < 60 kg. The e-agent detected 73.4% and 75.5% of those cases. Multiple absolute overdoses were found in 204 patients. The e-agent detected 72.7% of those. 90 multiple overdoses occurred during the same hospital stay and 11 on consecutive days. *Conclusion* Paracetamol overdose is a common medication error in hospitalized patients, which may occur due to process failures such as wrong timing when changing administration route or when factors like comedication and low body weight are ignored. The e-agent detects cases of paracetamol overdose, and therefore, can help prevent this kind of medication error in the clinical setting.

## Impacts on practice

Electronic algorithms that extract information from clinical information systems can improve medication safety by allowing timely identification of medication errors.

This e-agent proved to be useful in detecting paracetamol overdosing with a good detection rate for paracetamol doses > 5 g/day or > 60 mg/kg/day.

In practice, there is limited awareness of the necessity of adjusting a paracetamol dose in the presence of factors that may result in paracetamol toxicity, as well as of the importance of correct timing when switching between drug dosage forms.

## Introduction

In hospital setting, Paracetamol (acetaminophen) is commonly prescribed for a wide range of acute or chronic clinical conditions [1]. According to the Summary of Products Characteristics (SmPC) of paracetamol containing drugs the recommended maximal single dose is 1000 mg or 15 mg/kg; the maximal daily dose is 4000 mg or 60 mg/kg/day in adults [2].

Paracetamol has a safer gastrointestinal profile and fewer drug interactions than non-steroidal anti-inflammatory drugs (NSAIDs), but in case of overdosing it is associated with increased risk of mortality as it can lead to hepatotoxicity [3]. A small amount of Paracetamol is metabolized into the livertoxic N-acetyl-p-benzoquinone imine (NAPQI), that must be detoxified by glutathione [1]. Glutathione depletion (caused by malnutrition, muscular atrophy, chronic ethanol consumption or hepatic diseases) and induction of CYP450 enzymes are associated with higher hepatic toxicity [4]. Paracetamol toxicity may occur at therapeutic doses in specific populations like alcoholics, patients with low body weight or with hepatic diseases [1, 4, 5]. Higher risk of toxicity is also observed in older and frail patients [6]. Even if the exact dose adjustment strategy for each condition is not well defined [7, 8], it is recommended to consider precaution or a dose reduction in patients with one or more risk factors for paracetamol hepatotoxicity [1, 2, 4, 6–9]. The SmPCs demand a dose reduction in patients with chronic liver disease combined with other risk factors or end-stage renal disease [2].

The use of clinical information systems (CIS) has led to more accurate prescriptions as well as reduced risk of medication errors (ME) and drug-related adverse effects [10, 11]. To exploit the CIS's potential to detect ME timely, an electronic tool with algorithm-based alerts (e-agent) for paracetamol overdosing was developed and implemented within the hospital's CIS. The automatic alerts were subsequently validated by an expert to prevent irrelevant warnings from bothering the physicians. The paracetamol e-agent was developed as the first step towards a multi-agent framework for the detection of ME.

### Aim of the study

This study investigated the performance (defined as detection rate) of an e-agent to automatically detect paracetamol overdosing. The secondary objective was to analyze the causes of overdose in a hospital setting.

### Ethics approval

This study was approved by the Ethics Committee Northwest and Central Switzerland (Project-ID: 2020–00,329).

## Method

This was a retrospective cohort study identifying all cases of paracetamol overdosing in a Swiss tertiary care hospital during a 12-month period (1.01—31.12.2017). The identified cases were compared to the cases that had been detected prospectively by an e-agent during the same period. This e-agent was operational in the hospital’s CIS (“KISIM”, designed by Cistec AG) since mid-2016. This CIS includes a clinical physician order entry (CPOE), an electronic medication administration record (eMAR) and the patient's medical record and history.

For the retrospective analysis, all patients 18 years or older who had received at least one dose of paracetamol in any formulation, either alone or in combination with other drugs were included. Patients which did reject a general consent to use their generated data during treatementwere excluded. Patients treated in the emergency department, in the intensive or intermediate care units were excluded as these units use another CPOE/eMAR software.

The following data was extracted from the CIS for included patients: details of paracetamol prescription (dose, route, schedule) and administration (dose, time, route), body weight measurements, age, entrance and discharge date, concomitantly prescribed hepatic enzyme-inducing drugs..) Duplicate paracetamol administration records were excluded and records containing an unfeasible value such as “1 mg”, “1000 g” or “1000 tablets” were corrected and adjusted to the most likely dose in line with the actual medication order. Weight measurements closest in time to paracetamol administrations were used to calculate relative dosing. The records were analyzed by an algorithm programmed in Excel 2010 using Visual Basic for Applications (VBA). The number and prevalence of patients with an absolute overdosing of > 4 g/day were calculated. In case of an absolute overdose, the dose received was calculated and the number of days with an overdose. The reason for overdosing was identified. For patients with a body weight < 60 kg, the number and prevalence of patients with relative overdosing of > 60 mg/kg/day were calculated.

**Fig. 1:**
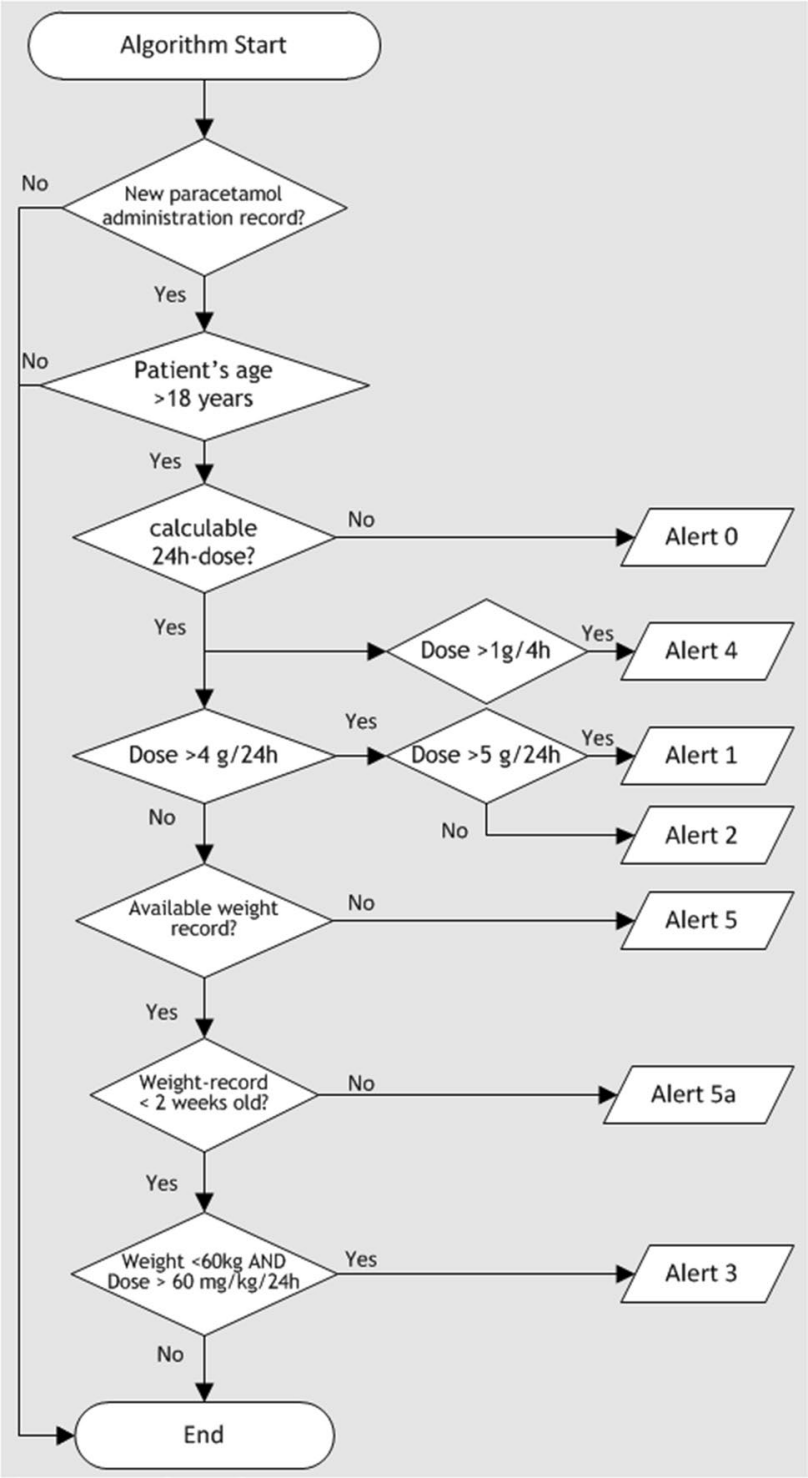
Visualisation of the alerts emitted by the algorithm-based electronic agent for paracetamol overdosing events^a^. ^a^Alert 0: dose no calculable; Alert 1: dose > 5 g/24 h; Alert 2: dose 4-5 g/24 h; Alert 3: dose > 60 mg/kg/24 h, Alert 4: dose > 1 g/4 h; Alert 5: no weight record available; Alert 5a: old weight record

- The e-agent checked the eMAR every 5 min for new administrations of paracetamol to adult patients (> 18 years) (Fig. 1). It calculated: the 24 h cumulative dose to detect absolute overdosing (> 4 g/24 h),
- the weight-based daily dose to detect relative overdosing (defined as > 60 mg/kg),
- the time between two administrated doses in order to detect too short intervals (< 4 h). This ME serves as indicator for absolute overdosing on the same day.

The e-agent generated different alerts if appropriate: incalculable dose; > 5 g/24 h; 4–5 g/24 h; > 60 mg/kg/24 h; > 1000 mg/4 h; no available weight data; old weight record (last available weight record older than 14 days).

The electronic agent summarized the alerts on a patient-by-patient basis, constantly adding the new alerts to those already existing for a specific patient. All alerts except “no available weight” were validated daily by a pharmacist (Monday to Friday). The validation of a > 60 mg/kg/24 h alert was restricted to patients with a body weight < 60 kg. This was carried out prospectively in 2017.

In cases with absolute overdosing, the pharmacist recorded the cause and, if appropriate, submitted an action proposal to the patient’s responsible physician In cases of relative overdosing, a dose reduction was recommended for patients < 50 kg. For patients weighing between 50 and < 60 kg, a dose reduction may have been recommended after evaluation of the clinical situation, in case of paracetamol hepatotoxicity risk factors (estimated glomerular filtration rate (eGFR) < 15 ml/min, chronic alcohol consumption, concomitant hepatic enzyme inducers, age > 75 years, severe hepatic impairment). The pharmacist recorded the outcome of the proposal and the time needed to process the alerts.

The number of action proposals submitted to physicians during 2017 was counted and the acceptance rate calculated.

The e-agent’s performance was defined as the percentage of patients receiving an absolute or relative overdose (identified by the retrospective study) for which the e-agent had emitted an alert (matched by the patient-case number). The total number of alerts during the study period as well as per day, per patient and per category was counted.

In contrast to the e-agent, the retrospective analysis calculated the absolute overdosing on a calendar-day basis and not on a 24 h basis.

In order to characterize patients receiving > 5 g/day or > 4 g over consecutive days as well as those weighing < 50 kg and receiving > 4 g for one day, the electronic medical records were reviewed by hand for chronic alcohol consumption, liver disease and laboratory parameters (eGFR < 15 ml/min, gamma-glutamyl transferase, liver transaminsases and bilirubin).

## Results

13,196 patients receiving at least one paracetamol dose were included. Of 234,418 administered doses, there were 25 different commercial preparations; 197,334 oral administrations (84.2%), 37,059 intravenous (15.8%), and 25 rectal (0.01%). A further 943 (0.4%) administrations were a combination of paracetamol and another drug.

During the study period the e-agent generated a total of 64,921 alerts (average 178 alerts/day) for 8108 patients (average 8 alerts/patient). The majority involved the categories “no available weight” (38.40%) and “ > 60 mg/kg/24 h” (27.78%, concerning 1610 patients). The alert “4–5 g/24 h” was recorded for 873 patients, and 128 patients were administered “ > 5 g/24 h”. Type of alerts and patient numbers are summarized in Table 1.

**Table 1:** Frequency of alerts by category (% of all generated alerts)

Category of alert	Number of alerts	Number of patients^a^
Alert 5: no weight record	24,930 (38.4)	4481
Alert 3: over 60 mg/kg/24 h	18,036 (27.8)	1610
Alert 5a: old weight record	11,978 (18.5)	2722
Alert 4: interval < 4 h	7818 (12.0)	1460
Alert 2: 4–5 g/24 h	1715 (2.6)	873
Alert 1: over 5 g/24 h	236 (0.4)	128
Alert 0: dose not calculable	208 (0.3)	51
Total alerts	64,921	

^a^Total number of patients: 8108; more than one alert may be generated for each patient

The pharmacist spent an average of 30 min/day validating the generated alerts and submitted 532 recommendations to the responsible physicians. The reasons for recommendations are summarized in Table 2, as well as the number of accepted recommendations. The majority of interventions involved relative overdosing (219 recommendations), maximum dose per 24 h exceeded (136), and relative overdosing in patients aged > 75 years (84). 61 patients were discharged within the next 24 h after the intervention and the result could not be properly assessed. The recommendations for the remaining 471 patients achieved an acceptance rate of 78%.

**Table 2:** Reasons for sending a recommendation to medical staff following agent alerts (% of all interventions)

Reason for intervention	Number of interventions	Accepted interventions
Overdose due to body weight	219 (41.2)	145
Maximum dose per 24 h exceeded	136 (25.6)	98
Overdose due to body weight and age	84 (15.8)	67
Lack of documentation	34 (6.4)	17
Overdose due to reserve	28 (5.3)	19
Overdose due to not scheduled administration	9 (1.7)	6
Overdose due to alcoholism	7 (1.3)	6
Overdose due to age	7 (1.3)	5
Overdose due to drug interactions	3 (0.6)	2
Overdose due to body weight and alcoholism	2 (0.4)	2
Other	2 (0.4)	1
> 5 g in 24 h in addition to Rifampicin	1 (0.2)	0
Total	532	368

### Absolute overdosing

The retrospective analysis of per calendar-day overdosing found 2524 absolute overdoses (> 4 g) in 2292 patients (17.4% of all receiving paracetamol) during the study period. 2276 patients were exposed to at least 1 day administration of 4–5 g paracetamol (the majority of these, 2204 patients, received exactly 5 g).

Thirty-nine patients (0.30% of total) received at least once > 5 g/day (35 of these received exactly 6 g paracetamol). No patient was administered > 6 g/day or consecutive administration of 6 g/day. Among patients with > 5 g/day, 12 were aged > 75, 3 suffered from alcoholism, 4 weighed < 60 kg, and 1 < 50 kg.

Of all paracetamol-receiving patients, only twenty-one patients were on hepatic enzyme inducers: 3 were given once > 4 g/day.

**Fig. 2:**
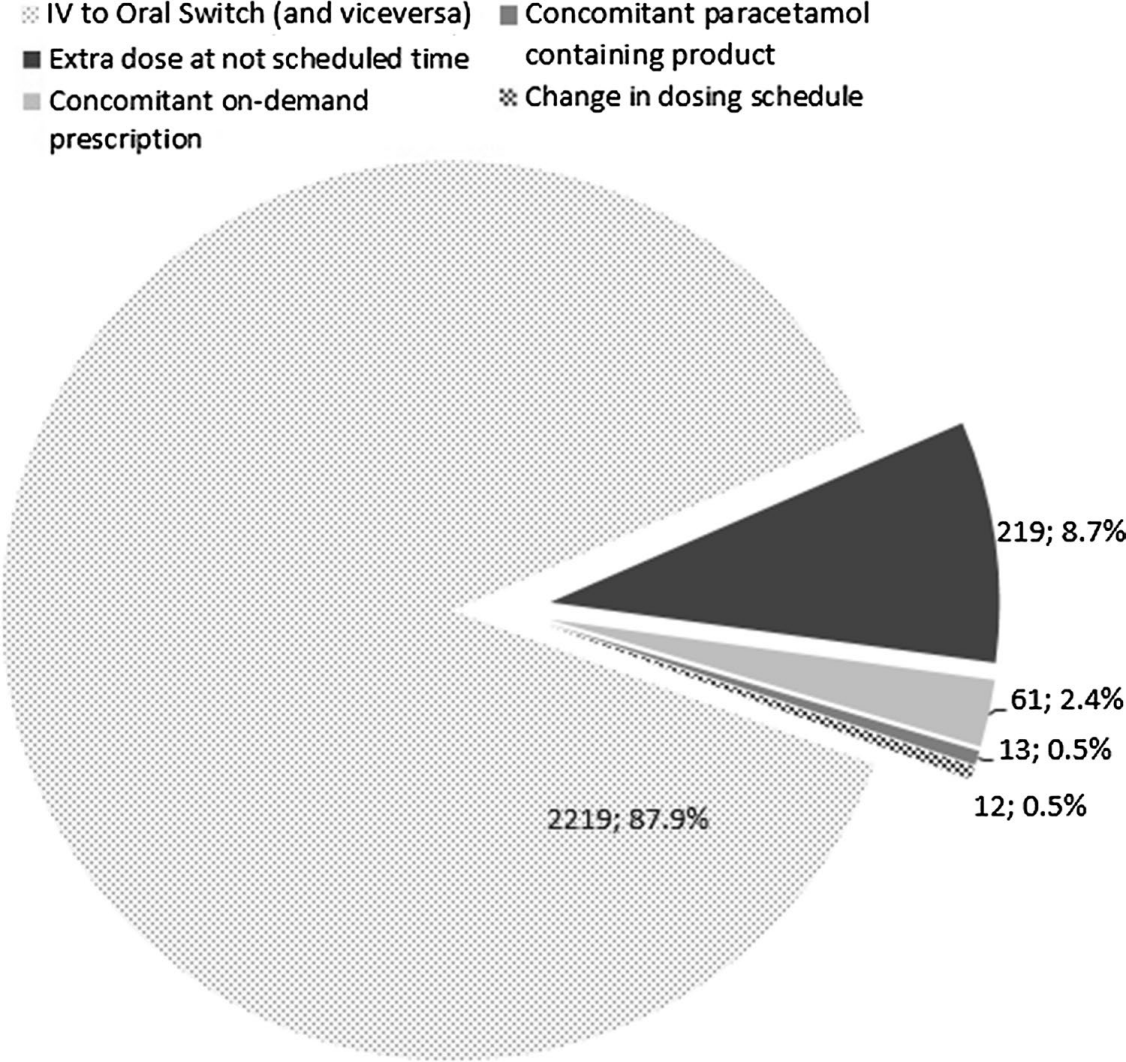
Causes of absolute overdosing according to the retrospective analysis

The causes of absolute overdoses according to the retrospective analysis are shown in Fig. 2. Most cases (87.9%) were attributable to a switch from intravenous to oral therapy, and 8.7% to an out-of-schedule dose without a standing on-demand order. Among the 39 patients with absolute overdosing of > 5 g on a calendar-day basis, the e-agent generated an alert for 34 patients (87.2%). Among the 2276 patients with a 4–5 g dose/calendar-day, it detected only 645 (28.3%). The performance of the e-agent in detecting per calendar-day overdosing is shown in Table 3.

**Table 3:** Comparing the number of patients detected by the electronic agent and actual number of affected patients

	Overdose > 5 g	Overdose > 4−5 g	> 4 g on consecutive days	Relative overdose in patients < 60 kg	Relative overdose in patients < 50 kg
Real data	39	2276	11	911	158
Reported by e-agent	34	645	8	688	116
e-agent performance	87.2	28.3	72.7	75.5	73.4

### Multiple absolute overdoses

**Fig. 3:**
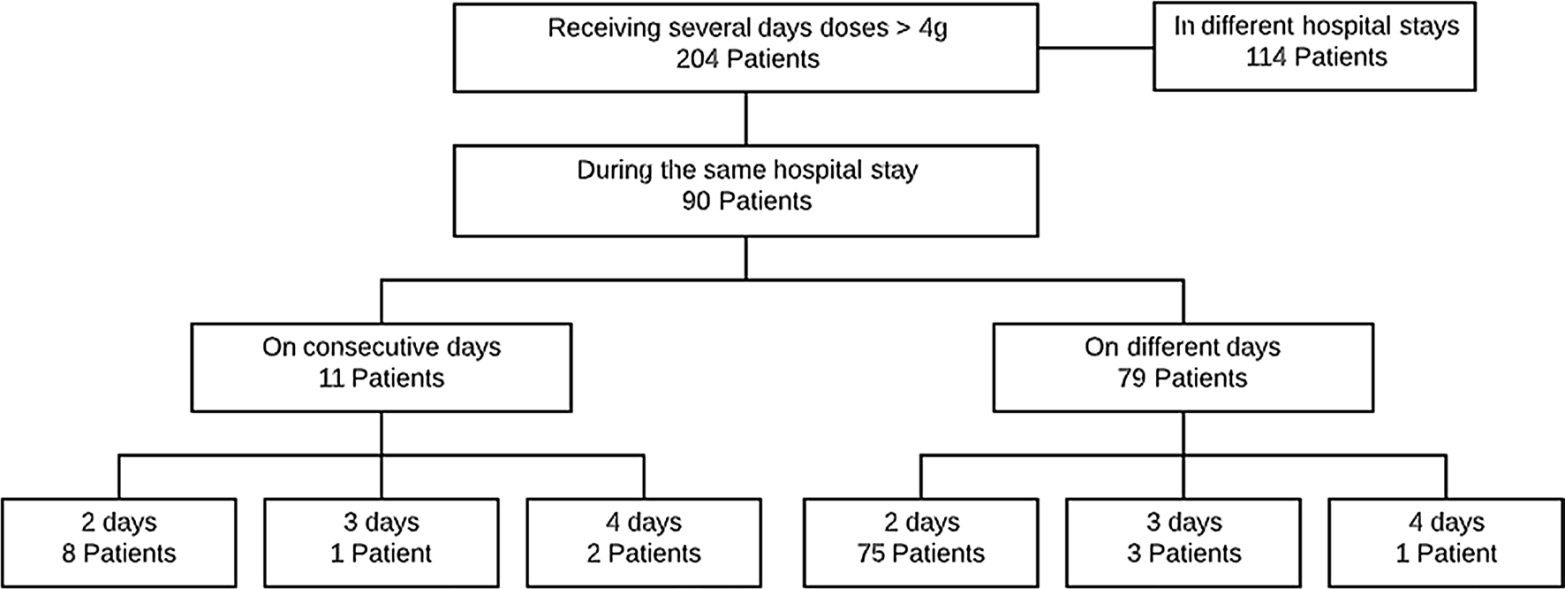
Patients with multiple absolute overdoses of paracetamol

In the retrospective analysis, multiple daily overdoses (> 4 g) were detected in 204 patients. Figure 3 shows the days of paracetamol overdosage in these patients. Consecutive overdosing 4–6 g/day (range 4300–5325 mg/day, 72.7% being 5 g) occurred in 11 patients, with an alert generated in 8 (72.7%). Regarding risk factors, only 1 patient was aged > 75; liver values were not available.

### Absolute overdose in patients with body weight less than 50 kg

In the next step absolute overdoses in patients weighing < 50 kg were analyzed. Of 525 patients weighing < 50 kg, 21 (4.0%) received a dose > 4 g/day and none on consecutive days. The e-agent generated an appropriate alert for only 6 (28.57%) of these 21 patients. In this group, 5 patients were older than 75, 2 had a slight increase in liver enzymes, and 1 had a transient increase approximately three times the initial measured value (on second day post-overdose).

### Relative overdosing based on patients’ weight

**Fig. 4:**
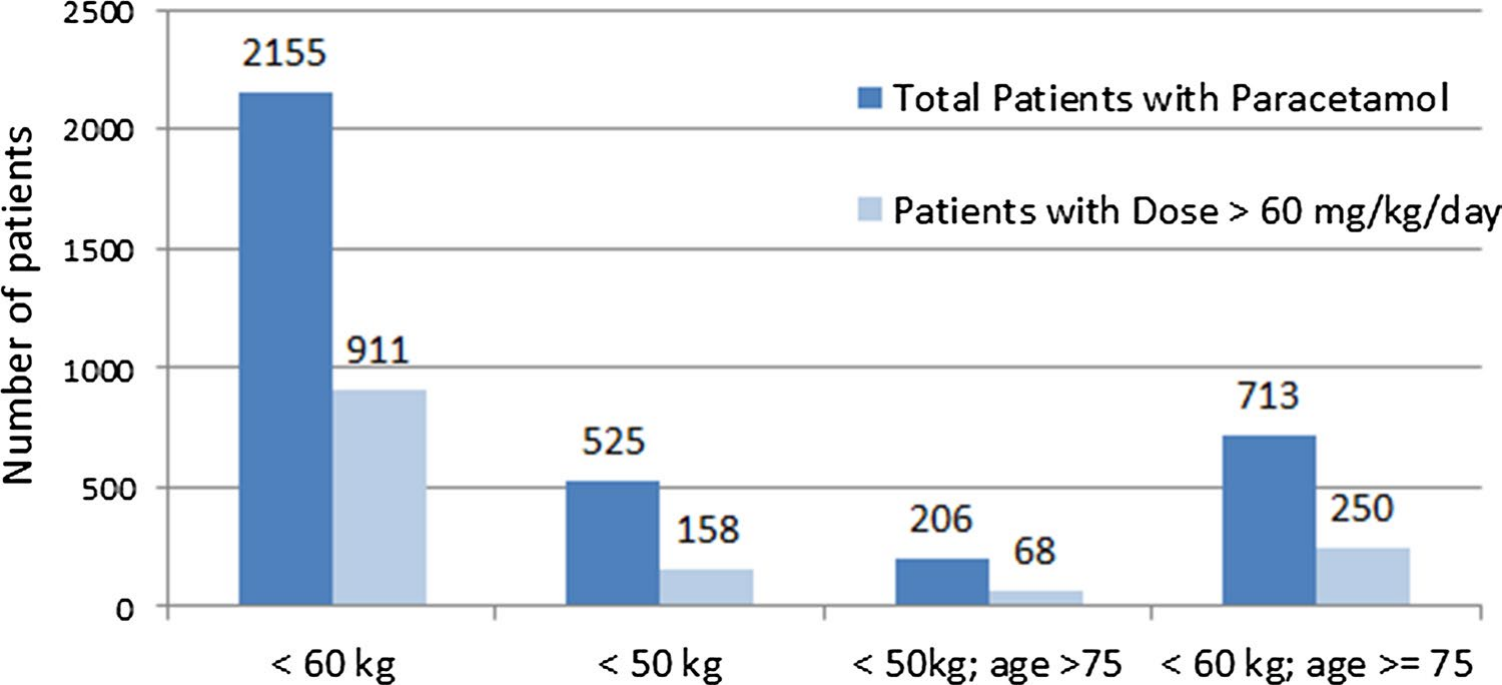
Relative overdosing in patients with a body weight < 60 kg and age  ≥75

A record of weight was available for 12,169 patients (92.2%) receiving paracetamol. 2155 of these (17.71%) weighed < 60 kg and 525 (4.31%) < 50 kg. The maximal daily dose of 60 mg/kg was exceeded in 911 (42.27%) and 158 cases (30.1%), respectively (Fig. 4). The e-agent created a relative overdosing alert for 116 patients (73.4%) weighing < 50 kg, and for 6 88 patients (75.5%) < 60 kg (Table 3).

## Discussion

In the present study, out of 13,196 hospitalized patients, 39 patients had a paracetamol overdose of > 5 g/day and 2276 had slight overdoses (4–5 g/day). The main reason found for overdose > 4 g/day was a switch from one application route to another without considering the time of last administration. This is in accordance to another Swiss study looking at paracetamol overdosing in hospital setting [12]. Accordingly, overdosing was in the majority of cases a single day event and happened rarely on consecutive days. In another 8.7% of cases, an out-of-schedule dose was given without a standing on-demand order, almost always following an insufficient analgesia during the night It could not be evaluated whether this was done with or without oral consultation with the doctor, or as an early morning dose with lack of subsequent communication. These findings not only highlights the importance of administering medication strictly according to physician prescription in hospitals, but also shows the importance of instructing medical staff about correct timing.

Concomitant on-demand prescription or a second paracetamol-containing product occurred in only < 2.9% of all cases, indicating that duplicate paracetamol prescription is rarely the cause of absolute overdosing in a hospital setting. Still, it should not be ignored because this kind of ME is susceptible to cause multiple overdoses.

Of all patients weighing < 50 kg, approximately 30% of adults and 33% of those > 75 years received > 60 mg/kg/day. As they may be malnourished and have depleted glutathione stores they may be at a greater risk for toxicity [13]. Previous reports have found that paracetamol overdose can be critical in older individuals [14, 15] as they have decreased capacity for metabolism and regeneration, as well as altered clinical pharmacokinetics of paracetamol [16] and as they may have several risk factors for paracetamol toxicity.

These findings show the importance of increasing physician knowledge about the necessity of adjusting paracetamol dose in adult underweight patients.

Paracetamol is generally prescribed without assessing the liver enzymes. Preexposure hepatic parameters were only available for 13 cases of an overdose > 5 g or on consecutive days. Postexposure values were available in 5 instances. No relevant increase or signs of hepatotoxicity were found.

For the retrospective analysis, the dose per 24 h was calculated on a calendar-day basis, whereas the e-agent calculated the 24 h cumulative dose after every new administration.

In daily practice, minor changes in real and scheduled administration time for regularly prescribed paracetamol are common. This was noticed after the implementation of the e-agent and led to a high number of false positive alerts of the type 2 (4–5 g/24 h) and of the type 4 (interval < 4 h). This problem is less frequent using a calender-day basis (midnight doses are rare) and was considered in the programming for the retrospective analysis in order to exclude artificial overdoses.

Compared to results of the retrospective analysis, the e-agent identified 87.2% of "per calendar day" overdoses > 5 g and 72.7% of overdose > 4 g on consecutive days. It was, however, only able to detect 28.3% of 4–5 g overdoses. The main cause—the documented time of administration—for this lower performance was identified in the retrospective analysis: in the eMAR of the study hospital it is possible to record an administration 60 min in advance. As the e-agent looks for new paracetamol records every 5 min, it detects the new record but, as it has not been administered yet, does not add the dose to the calculation of the absolute dose in the last 24 h. This problem will beconsidered in the programming of new e-agents. Second, the category “too short interval” (> 1000 mg/4 h) overshadowed the alerts of the categories 4–5 g/24 h and > 60 mg/kg/day, preventing the latter from being displayed.

The e-agent successfully detected 75.5% and 73.4% of relative overdose (> 60 mg/kg/day) in patients < 60 kg and < 50 kg, respectively. Two problems were responsible for this lower efficacy rate compared to the absolute overdoses > 5 g: firstly, patients with a weight record older than 14 days were categorized under “old weight record” and the relative dose was not calculated. (The weight was displayed in the alert, so the pharmacist could process it, but there was a risk to overlook relevant cases.) Secondly, as identified in the retrospective study, if a scheduled weight measurement was not performed, the e-agent did not check for previous measurements, but instead emitted a “no available weight” alert. Both issues will be corrected in a new version of the e-agent.

An earlier study conducted in a Swiss university hospital using the same CIS analyzed the efficacy of a similar alert system combining automated detection with subsequent expert validation. They reported absolute overdosing (> 5 g/day) in 0.4% of patients, 91.3% of which were detected by the alert e-agent [12]. The diagnostic accuracy of the e-agent in this study was lower, but (in contrast to this one) their system did not look for relative overdosing or short intervals. Secondly, their e-agent did not provide "real-time alerts" but was based on a once daily record extraction. The higher complexity of this e-agent alert and its real-time implementation could explain the lower performance. It does indicate, however, that the developed and studied alert system requires several optimizations.

The e-agent analyzed in this study generates too many alerts to bypass the validation by a pharmacist. Too many not relevant alerts of an electronic system can cause the physician to ignore and override them [17]. In this study, an acceptance rate of > 70% was achieved. The current e-agent is not yet highly specific and therefore still requires modification before it can be automated.

The present study has several limitations, related to study design and shortcomings of the alert system. Firstly, the accuracy of the alert system depends on clinical documentation. Undocumented cases can't be processed and any errors in data entry could also affect results. In addition, as it is a commonly used medication, patients might have used paracetamol-containing drugs without it being documented. On the other side, paracetamol overdoses have been prevented, usually by the alert 4 ("interval < 4 h."). This study did not compare the incidence of paracetamol overdose before and after the implementation of the e-agent, so a potential reduction could not be calculated.

Nevertheless, this study confirms the usefulness of such an electronic tool to detect ME and this e-agent can be considered as a successful proof of concept for the development and integration of other algorithms into a CIS. Paracetamol was used as first e-agent due to its high prescription rate, which made it possible to identify relevant aspects for the development of other e-agents.

## Conclusion

The results of the present study showed that paracetamol overdose is prevalent in hospitalized patients. It may occur due to timing errors, especially when switching between dosage forms or administration routes, or other causes like ignoring underweight, which must receive more attention from physicians. This electronic agent with algorithm-based alerts could detect cases of paracetamol overdose and can be, after some optimizations, recommended in the clinical setting to minimize this medication error.
